# Genetic analysis of basal stalk rot resistance introgressed from wild *Helianthus petiolaris* into cultivated sunflower (*Helianthus annuus* L.) using an advanced backcross population

**DOI:** 10.3389/fpls.2023.1278048

**Published:** 2023-10-18

**Authors:** Zahirul I. Talukder, William Underwood, Christopher G. Misar, Xuehui Li, Gerald J. Seiler, Xiwen Cai, Lili Qi

**Affiliations:** ^1^ United States Department of Agriculture (USDA)-Agricultural Research Service, Edward T. Schafer Agricultural Research Center, Fargo, ND, United States; ^2^ Department of Plant Sciences, North Dakota State University, Fargo, ND, United States; ^3^ Wheat, Sorghum and Forage Research Unit, United States Department of Agriculture (USDA)-Agricultural Research Service, Lincoln, NE, United States

**Keywords:** sunflower, *Helianthus petiolaris*, *Sclerotinia sclerotiorum*, basal stalk rot resistance, quantitative trait loci (QTL), QTL mapping

## Abstract

**Introduction:**

*Sclerotinia sclerotiorum* is a serious pathogen causing severe basal stalk rot (BSR) disease on cultivated sunflower (*Helianthus annuus* L.) that leads to significant yield losses due to insufficient resistance. The wild annual sunflower species *H. petiolaris*, commonly known as prairie sunflower is known for its resistance against this pathogen. Sunflower resistance to BSR is quantitative and determined by many genes with small effects on the resistance phenotype. The objective of this study was to identify loci governing BSR resistance derived from *H. petiolaris* using a quantitative trait loci (QTL) mapping approach.

**Methods:**

BSR resistance among lines of an advanced backcross population (AB-QTL) with 174 lines developed from a cross of inbred line HA 89 with *H. petiolaris* PI 435843 was determined in the field during 2017-2019, and in the greenhouse in 2019. AB-QTL lines and the HA 89 parent were genotyped using genotyping-by-sequencing and a genetic linkage map was developed spanning 997.51 cM and using 1,150 SNP markers mapped on 17 sunflower chromosomes.

**Results and discussion:**

Highly significant differences (*p*<0.001) for BSR response among AB-QTL lines were observed disease incidence (DI) in all field seasons, as well as disease rating (DR) and area under the disease progress curve (AUDPC) in the greenhouse with a moderately high broad-sense heritability (*H* ^2^) of 0.61 for the tested resistance parameters. A total of 14 QTL associated with BSR resistance were identified on nine chromosomes, each explaining a proportion of the phenotypic variation ranging from 3.5% to 28.1%. Of the 14 QTL, eight were detected for BSR resistance in the field and six were detected under greenhouse conditions. Alleles conferring increased BSR resistance were contributed by the *H. petiolaris* parent at 11 of the 14 QTL.

## 1 Introduction

Sunflower (*Helianthus annuus* L.) is the third most important oil-producing crop after soybean and rapeseed, and global sunflower-seed production is forecasted to reach 54.3 million tons in the 2023–2024 growing season, an increase of 6% from the previous year (USDA, 2023). Approximately 86% of the U.S. sunflower is grown in the states of North Dakota, South Dakota, and Minnesota (USDA, 2022). The cool and humid climatic conditions during the summer growing season of this region are highly favorable for sunflower diseases caused by the fungus, *Sclerotinia sclerotiorum* (Lib) de Bary. *Sclerotinia sclerotiorum* is a soilborne necrotrophic fungal pathogen with a wide host range, including many important broadleaf food crops (Boland and Hall, 1994; Bolton et al., 2006). The fungus causes several distinct forms of disease on various parts of the sunflower plant throughout its life cycle. These include root infection leading to basal stalk rot (BSR) lesions near the soil line, mid-stalk rot (MSR) resulting in lesions in the middle of the stalk, and head rot (HR) affecting the capitulum (Gulya et al., 1997). The sclerotia of the fungus overwinter in the soil and in plant debris, and serve as the primary source of inoculum in the field. Under favorable growth conditions, the overwintering sclerotia beneath the soil undergo either myceliogenic or carpogenic germination to incite sunflower diseases (Gulya et al., 2019). Mycelial germination of the sclerotia causes wilt and BSR disease when hyphae directly infect sunflower roots, while carpogenic germination results in the production of apothecia and airborne ascospores, infecting sunflower heads and stems to cause HR and MSR symptoms, respectively (Gulya et al., 1997). Head rot and BSR have been considered as the most damaging Sclerotinia diseases in the United States (Gulya et al., 1989; Underwood et al., 2021). During an epidemic year, such as occurred in 1986, the outbreak of the two diseases in the U.S. sunflower crops caused hundreds of millions of dollars in losses (Gulya et al., 1989).

Sclerotinia BSR disease in sunflower routinely causes serious economic losses in the northern Great Plains states North and South Dakota, and Minnesota (Seiler et al., 2017a; Gulya et al., 2018). Unlike other hosts, BSR management in sunflower using foliar fungicidal sprays is ineffective due to the unique belowground myceliogenic root infection (Seiler et al., 2017a). The utilization of host genetic resistance is generally regarded as the most economical and environmentally friendly approach to manage BSR disease in sunflowers. The genetics of host resistance against *S. sclerotiorum* is quantitative and governed by many small effect genes (Bert et al., 2002; Davar et al., 2010; Talukder et al., 2016; Talukder et al., 2021; Talukder et al., 2022). No complete resistance against Sclerotinia diseases has been identified in the sunflower gene pools. Increased Sclerotinia resistance has been achieved through routine mining of novel resistance sources in the sunflower primary gene pool and incorporated into breeding programs (Miller and Gulya, 1999; Talukder et al., 2014a; Seiler et al., 2017a; Talukder et al., 2017; Hulke et al., 2018; Koehler et al., 2019; Money et al., 2019; Smart et al., 2019). Efforts have been made to study the nature and magnitude of BSR resistance alleles conferring quantitative genetic variations within sunflower germplasms using recombinant inbred line (RIL) populations, and a total of 20 quantitative trait loci (QTL) associated with BSR resistance were identified from two RIL populations developed from the crosses of HA441/RHA349 and PAC2/RHA266. These QTL are distributed on 13 of the 17 sunflower chromosomes (**Supplementary Table S1**, Davar et al., 2010; Amouzadeh et al., 2013; Talukder et al., 2014b; Talukder et al., 2016; Paknia et al., 2020).

Cultivated sunflower apparently evolved through the domestication processes from a limited number of wild ancestors, a consequential process that fundamentally shrinks the genetic variability in the crop gene pool compared to its wild progenitor (Ladizinsky, 1985). Successive human activity, for example, selective intercrossing followed by intensive selection for elite crop varieties, has further narrowed the gene pool in many crops. The depleted genetic diversity within the elite cultivars not only makes them vulnerable to diseases, but also decreases the possibility of finding suitable genes to combat new virulent pathogenic races. Sunflower is no exception to this phenomenon (Cheres and Knapp, 1998), with breeders encountering difficulties in breeding for BSR resistance in sunflower because of limited known sources of resistance. Sunflower originated and was initially domesticated in eastern North America (Blackman et al., 2011) with its wild relatives (14 annual and 39 perennial species) adapted to a wide range of habitats and serve as potential sources of novel genes for a number of economically important diseases (Seiler, 2010; Seiler et al., 2017b). To broaden the genetic diversity for Sclerotinia resistance, attempts have been made to discover useful resistance sources for breeding and for genetic studies from the wild annual species. High levels of BSR resistance were identified in the wild annual species *H. argophyllus* Torr. & Gray*, H. praecox* Engelm. & Gray, and *H. petiolaris* Nutt. (Block et al., 2010; Block et al., 2011), and several introgression lines have been released with higher levels of BSR resistance that both private and public breeders can deploy in developing commercial hybrids (Qi et al., 2016; Qi et al., 2018; Talukder et al., 2019a; Talukder et al., 2019b). The genetic basis of BSR resistance in some of these introgression lines has been studied to identify QTL associated with BSR resistance using advanced backcross QTL (AB-QTL) mapping populations (Tanksley and Nelson, 1996) to discover DNA markers closely associated with QTL to make breeding and selection more efficient (**Supplementary Table S1**, Talukder et al., 2021; Talukder et al., 2022).

The wild annual sunflower *H. petiolaris* is a diploid species commonly known as the prairie sunflower and grows throughout the Plains region of the central United States (Rieseberg et al., 1999). The species is a valuable resource of many economically important genes and traits that have been transferred into cultivated sunflower, including cytoplasmic male sterility (CMS PET1, Leclercq, 1969), sunflower rust resistance (Jan et al., 2004; Qi et al., 2011), and resistance to the sunflower moth (Rogers et al., 1984). *Helianthus petiolaris* has also been previously reported to possess high levels of resistance against Sclerotinia diseases (Cáceres et al., 2006; Block et al., 2010; Gutierrez et al., 2012). The objectives of this study are to identify QTL associated with Sclerotinia BSR resistance derived from *H. petiolaris* using an AB-QTL population and to link these QTL to SNP markers that can be used for selecting lines with higher levels of Sclerotinia BSR resistance in sunflower.

## 2 Materials and methods

### 2.1 Mapping population development

An advanced backcross QTL (AB-QTL) mapping population was used to study the BSR resistance introgressed from the wild annual sunflower *H. petiolaris* into cultivated sunflower. The AB-QTL population was composed of 174 lines developed by crossing and backcrossing of two cultivated sunflower inbred lines, HA 89 and HA 458, with the *H. petiolaris* subsp. *fallax* Heiser accession, PI 435843. A nuclear male sterile (NMS) HA 89 (PI 559477) (Jan and Rutger, 1988) was used to make the initial cross with the *H. petiolaris* accession in the greenhouse in 2009, followed by the cross with sunflower inbred line HA 458 (PI 655009), and the derived hybrids from this cross were treated as BC_1_ to simplify the pedigree. The subsequent backcross was made with the normal, male-fertile HA 89 (PI 599773) to produce the BC_2_ population. Both HA 89 and HA 458 are sunflower inbred maintainer lines with good agronomics but susceptible to BSR disease. HA 458 harbors the downy mildew resistance gene *Pl_17_* (Qi et al., 2015). The wild sunflower *H. petiolaris* accession PI 435843 collected from New Mexico is an annual diploid (2n=2x=34) (Qi et al., 2016). The *H. petiolaris* accession showed high levels of BSR resistance in the greenhouse tests (Block et al., 2010). F_1_ hybrids and BC_1_ and BC_2_ families were initially screened for BSR resistance in the greenhouse to select resistant individuals for advancement, while BC_2_F_3_ families were subsequently evaluated for BSR resistance in the field to confirm resistance identified in greenhouse trials (Qi et al., 2016).

### 2.2 Phenotypic assessment

#### 2.2.1 Field BSR evaluations

The *S. sclerotiorum* inoculum preparation for BSR evaluation trials was previously described by Underwood et al. (2021). A moderately aggressive *S. sclerotiorum* isolate, NEB-274, was used for all field and greenhouse screening trials. The AB-QTL population of the BC_2_F_2_-derived BC_2_F_4_ along with the cultivated sunflower parental line, HA 89, was evaluated for BSR resistance in inoculated field trials at the Carrington Research Extension Center, North Dakota State University, Carrington, ND during the 2017–2019 growing seasons. Two sunflower hybrids, Cargill 272 and Croplan 305, were used as susceptible and resistant checks, respectively, in each of the field trials. The experimental design was a randomized complete block design (RCBD) with three replications in each year. Seeds (30 per line) were planted in 6-m single-row plots with a row spacing of 75 cm. At the V-6 growth stage (Schneiter and Miller, 1981), the trials were inoculated with *S. sclerotiorum*-infested millet (*Panicum miliaceum* L.) inoculum. The inoculum (90 g/row) was side dressed in furrows using a tractor drawn granular chemical applicator at 5–7 cm depth and 15–20 cm away from each row (Gulya et al., 2008). Plants were evaluated for typical BSR symptoms of basal stem lesions at approximately 10 weeks after inoculation at physiological maturity and disease incidence (DI) was scored as the percentage of plants in a plot exhibiting BSR symptoms.

#### 2.2.2 Greenhouse BSR evaluations

The AB-QTL mapping population was evaluated in the greenhouse following the method described by Underwood et al. (2021). Briefly, the experimental design was an RCBD with three replications conducted in the winter of 2018–2019. Initially, 20 seedlings were grown from each of the BC_2_F_4_ families along with HA 89, one of the cultivated sunflower parents, and RHA 801, a BSR-resistant inbred line. Seeds were planted in 32-position sheet pots filled with potting mix (Metro Mix 902, Sun Gro Horticulture, Agawam, MA). Single seeds were planted in each pot and grown in the greenhouse at ~20°C with a 16-h photoperiod maintained by supplemental lighting. After 5 weeks, 12 healthy plants from each progeny family, parent, and resistant check were inoculated by removing root-bound plants, placing 0.76 g of *S. sclerotiorum*-infested millet inoculum in the bottom of the pot, and returning the plants to the pot to place roots in contact with millet inoculum (Underwood et al., 2021). Plants were then monitored daily for 28 days for death due to BSR and the number of days to plant death was noted for each individual.

### 2.3 Phenotypic data analysis

BSR DI data were collected for sunflower plants in the field trials at the physiological maturity stage. DI of each sunflower line was calculated as the percentage of plant showing typical BSR disease symptom in each row plot. In the greenhouse trial, data for BSR evaluation were collected for area under the disease progress curve (AUDPC) and days to death from BSR, referred to as disease rating (DR), following the method described by Underwood et al. (2021). Prior to performing an analysis of variance (ANOVA), all collected data were tested for normality using the Shapiro–Wilk test. Analyses of variance were performed using PROC MIXED in SAS version 9.4 (SAS Institute, 2016) where the effect of genotype was considered fixed while all other factors were considered random effects in the model. Broad-sense heritability (*H* ^2^) (entry mean basis) was estimated as described by Nyquist (1991) as: $H^{2}=\sigma_{g}^{2}/(\sigma_{g}^{2}+\sigma_{ge}^{2}/l+\sigma_{e}^{2}/r)$ for the field trials and $H^{2}=\sigma_{g}^{2}/(\sigma_{g}^{2}+\sigma_{e}^{2}/r)$ for the greenhouse trial, where $\sigma_{g}^{2}$ is the genetic variance, $\sigma_{ge}^{2}$ is the genotype × year variance, $\sigma_{e}^{2}$ is the error variance, *r* is the number of replications, and *l* is the number of years. Spearman rank correlation (ρ) analyses were performed using R version 4.1.1 (R Core Team., 2021) for all BSR disease data among lines of the HA 89/*H. petiolaris* AB-QTL population collected in the field during the 2017–2019 seasons and greenhouse in 2018–2019 winter.

### 2.4 Genotyping of the AB-QTL population

Leaf tissues of the AB-QTL BC_2_F_2_ population along with the parent HA 89 were collected from ~4-week-old sunflower seedlings and freeze-dried. Approximately 50 mg of freeze-dried leaf tissue per individual was used for isolation of genomic DNA using a Qiagen DNeasy 96 plant kit (Qiagen, Valencia, CA, USA) with modification of the manufacturer protocol as described by Horne et al. (2004). A NanoDrop 2000 Spectrophotometer (Thermo Fisher Scientific) was used to measure the quality and quantity of extracted DNA. Single-nucleotide polymorphism (SNP) genotyping was performed using the genotyping-by-sequencing (GBS) method described by Elshire et al. (2011) with minor modifications. Sequencing was performed on an Illumina HiSeq 2500 (Illumina, San Diego, CA) at the Genomic Sequencing and Analysis Facility at the University of Texas Southwestern Medical Center at Dallas, Texas. The GBS library preparation, sequencing, and genotype calling procedures have been described elsewhere (Talukder et al., 2021). Sequence alignment to the sunflower reference assembly HanXRQr1.0 (Badouin et al., 2017) provided a total of 55,318 SNP markers. SNP markers were named by assigning a “C” prefix followed by the chromosome number corresponding to one of the 17 sunflower chromosomes and a number identifying the physical position of the marker based on the HanXRQr1.0 reference genome assembly. A 400-bp sequence flanking each SNP found to be significantly associated with BSR resistance in this study is presented in **Supplementary Table S2.**

### 2.5 Linkage analysis

Prior to linkage analysis, the SNP markers were filtered to exclude monomorphic SNPs, missing genotype for the cultivated sunflower parent, >20% missing genotype, and markers exhibiting segregation distortion, resulting in a filtered dataset containing 2,157 markers for linkage mapping. JoinMap 4.1 software (Stam, 1993; Van Ooijen, 2006) was used to develop a linkage map for the AB-QTL population. In the software, the “‘similarity of loci” option was first chosen to identify co-segregating SNP markers with identical genotype (similarity value = 1), which mapped in the same loci. These “similarity of loci” markers were temporarily removed until the initial linkage map was constructed to reduce the time and computational burden of the linkage analysis. Independence logarithm of the odds (LOD) was chosen to assign markers to linkage groups (LGs) with threshold values ranging from 3.0 to 10.0. Linkage analysis and marker ordering were performed using the regression mapping algorithm, and the Kosambi mapping function was used to convert recombination fractions to map distances in centimorgans (cM; Kosambi, 1943). The SNP markers were subsequently mapped to 17 LGs that correspond to the 17 sunflower chromosomes. In the final linkage map, co-segregating similarity-loci markers that were previously excluded were added to the map in their corresponding positions.

### 2.6 Quantitative trait loci analysis

QTL analysis of the BSR DI data from the field trials was analyzed both for individual years and 3 years combined data using estimated best linear unbiased predictor (BLUP). Initial analysis was performed using WinQTL Cartographer v2.5 with the composite interval mapping (CIM) option (Zeng, 1994; Wang et al., 2012). The forward and backward regression method (model 6) of the CIM analysis was used to scan the sunflower genome for BSR resistance QTL. The parameters were set to select up to five control markers using a window size of 10 cM and a walk speed of 1 cM for the analysis. A total of 1,000 permutation tests were performed for each trait independently to declare significance LOD thresholds (Churchill and Doerge, 1994). The QTL analysis results from WinQTL Cartographer were validated by performing QTL analysis with two other popularly used software including QGene v4.3 (Joehanes and Nelson, 2008) and QTL IciMapping v4.1 (Meng et al., 2015). These two software packages provide similar algorithms for QTL scanning but different options for cofactor selection. QTL detected with at least two software packages with significant LOD values in the same genomic region were reported in this study. The QTL flanking region was estimated by calculating the 1-LOD drop support interval on either side of the most likely QTL peak position where the LOD score declined by 1 unit. MapChart v2.2 (Voorrips, 2002) was used to draw the linkage map displaying the detected QTL. The naming of the BSR resistance QTL identified in this study followed the convention detailed in Talukder et al. (2016). Each QTL name started with a prefix Q for QTL, followed by a three-letter descriptor of the trait (BSR), the LG number, and a sequential number denoting the total number of QTL identified to date on that LG for the specific trait.

## 3 Results

### 3.1 BSR resistance assessment in the field

**Figure 1:**
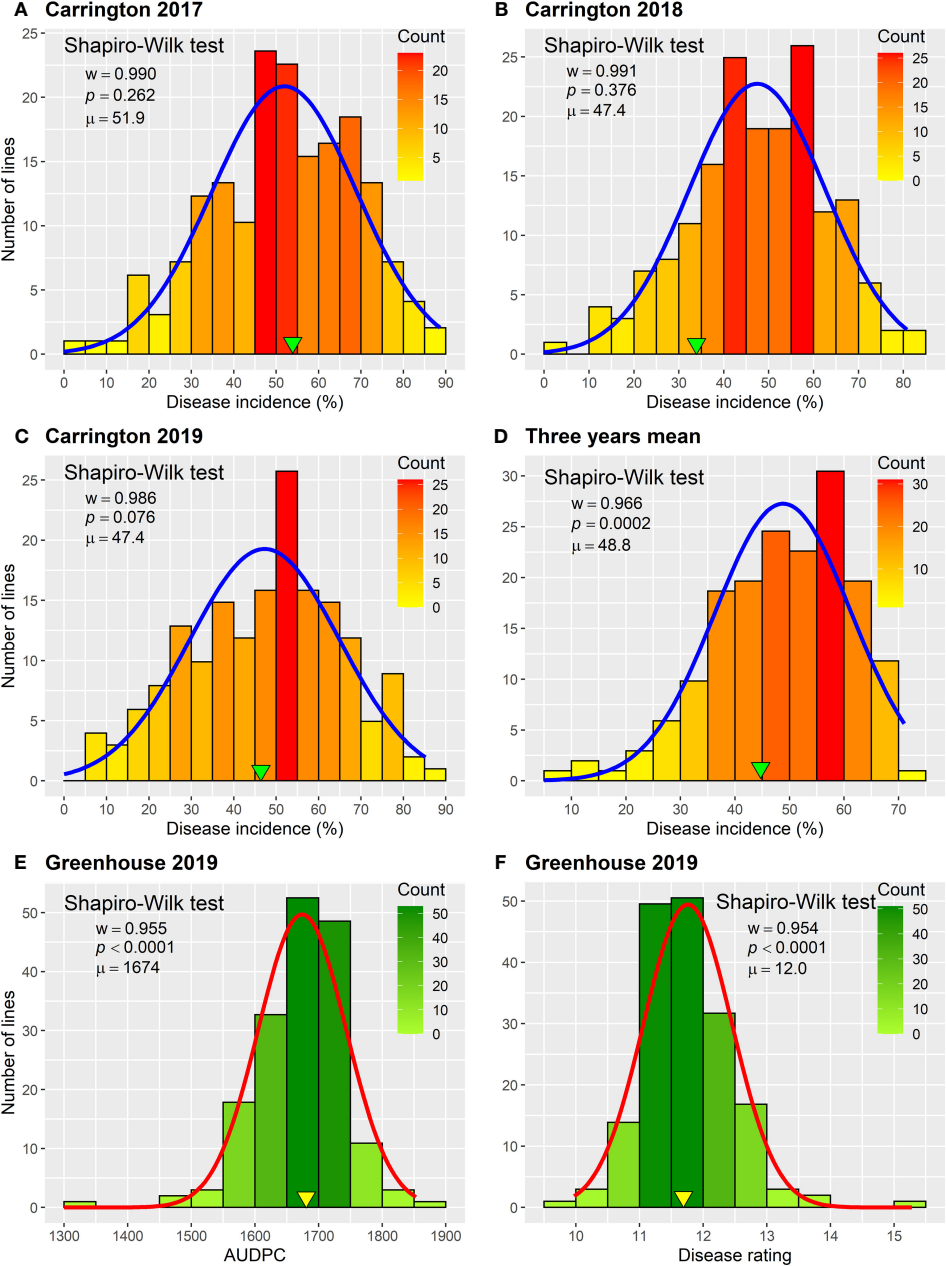
Frequency distributions of Sclerotinia BSR disease response parameters observed for the HA 89/*H. petiolaris* AB-QTL population in greenhouse and field trials. **(A–D)** Disease incidence observed in field trials conducted in Carington, ND during 2017–2019. **(E)** Area under the disease progress curve and **(F)** disease rating observed in greenhouse trials in 2019. Arrowheads indicate observed values for the cultivated sunflower parent HA 89. The Shapiro–Wilk normality test statistic (*w*), probability value (*p*), and population mean (μ) for each environment are displayed within the respective plots.

The HA 89/*H. petiolaris* AB-QTL BC_2_F_4_ population was evaluated for BSR resistance at Carrington, ND for three consecutive summer seasons during 2017–2019. The occurrence of BSR disease was very similar across the three growing seasons with the population mean BSR DI of 51.9% for 2017 and 47.4% for both 2018 and 2019 (**Figure 1**; **Supplementary Figure 1**). The progeny comprising the AB-QTL population showed a wide variation of BSR DI in all three seasons with a range of 4.8%–88.6%, 2.2%–80.8%, and 5.6%–85.2% for 2017, 2018, and 2019 Carrington trials, respectively. Overall, the combined mean BSR DI of the AB-QTL population across three growing seasons at the Carrington, ND location was 48.8%, ranging from 8.3% to 71.1%. Shapiro–Wilk test showed that the distribution of BSR DI data for all three seasons was normal with most progeny lines exhibiting an intermediate phenotype, while a few individuals showed extreme trait values (**Figure 1**). However, the distribution of the pooled BSR DI data for all three seasons was not normal and was skewed toward lower DI values (**Figure 1**). The BSR DI of the cultivated sunflower parent, HA 89, was 53.9%, 33.9%, and 46.4% for the 2017, 2018, and 2019 seasons, respectively, with a mean 44.7% across 3 years (**Figure 1**). Linear mixed model analysis of variance (ANOVA) of the BSR DI data showed highly significant (*p* < 0.001) genetic variations in all three seasons (data not shown). Highly significant (*p* < 0.001) genetic variation was also observed for the genotype in the combined analysis (**Table 1**). However, no significant effects of the environment or the genotype × environment (G×E) interactions were observed for the trait, indicating that the genotypes responded similarly across the 3 years of field trials. The broad-sense heritability (*H* ^2^) estimate was 0.61 for the trait measured in the field across 3 years. Correlations (ρ) among BSR DI observations across 3 years of field evaluations were highly significant (*p* < 0.001), validating the efficient repeatability of the BSR field trials across years (**Table 2**). We note that error variance accounted for a large proportion of overall variance in the linear mixed model analysis, highlighting the substantial variability in field trials for this disease that cannot be accounted for by blocking factors. This observation is consistent with previous studies involving similar field trials for BSR response and, in part, this variability in field trials for BSR prompted the development and parallel use of the greenhouse evaluation (Talukder et al., 2021; Underwood et al., 2021; Talukder et al., 2022).

**Table 1:** Combined analysis of variance (ANOVA) for Sclerotinia basal stalk rot (BSR) disease incidence (DI) scores among sunflower lines (BC_2_F_4_) derived from the cross of HA 89 and the wild annual sunflower species, *H. petiolaris*, in field trials at Carrington, ND during 2017 to 2019.

Component	df	Variance estimate	Confidence limit (0.05)		*F*/*Z* value†	*p*-value > *F*/*Z*
			Lower	Upper		
Gen	173	–	–	–	**2.55**	**<0.0001**
Env	2	$\sigma_{l}^{2}\;$ = 0.00	–	–	–	–
Rep (Env)	6	$\sigma_{r}^{2}\;$ = 48.71	21.44	197.95	1.89	0.0294
Gen × Env	345	$\sigma_{gl}^{2}\;$ = 20.19	6.718	234.46	1.28	0.1000
Error	1035	$\sigma_{e}^{2\;}$ = 489.37	449.77	534.48		

† Data were analyzed using the PROC MIXED model with genotypes considered as a fixed effect and, thus, subject to F-test (values indicated in bold). F, Fisher’s F-test statistic; Z, Z-test statistic; Gen, genotype; Env, environment.

**Table 2:** Spearman rank correlations (ρ) between Sclerotinia BSR data among lines of the HA 89/*H. petiolaris* AB-QTL population for field and greenhouse trials conducted from 2017 to 2019.

Environment		Carrington 2017	Carrington 2018	Carrington 2019	AUDPC
Field DI^1^	Carrington 2017	–	–	–	–
	Carrington 2018	0.34***	–	–	–
	Carrington 2019	0.24***	0.35***	–	–
Greenhouse	AUDPC	0.19*	0.04	0.04	–
	Disease rating	0.19*	0.04	0.04	1.00***

* Significant at p < 0.05, *** Significant at p < 0.001.

^1^DI, disease incidence.

### 3.2 BSR resistance assessment in the greenhouse

The frequency distribution of AUDPC and BSR DR data measured in greenhouse evaluations of the HA 89/*H. petiolaris* AB-QTL population also showed continuous quantitative variation (**Figure 1**; **Supplementary Figure 1**). The mean AUDPC of the HA 89 cultivated sunflower parent was 1,681, while the AB-QTL population mean was 1,674 ranging from 1,324 to 1,853 with a skewed trend toward higher values. The mean BSR DR of the HA 89 parent was 11.7 dpi (days post-inoculation) with a population mean of 12.0 dpi (range, 10.0 to 15.3 dpi). The DR data also showed a skewed distribution, but toward lower DR values. ANOVA for AUDPC and DR datasets from greenhouse trails also indicated highly significant (p < 0.001) genetic variations (**Table 3**). The broad-sense heritability (H^2^) was estimated at 0.62 for both AUDPC and DR traits. AUDPC and DR scores for the lines comprising the AB-QTL population were strongly and significantly correlated according to Spearman*’*s rank correlations (ρ = 1.00, p < 0.001) (**Table 2**). Comparison of the field and greenhouse screening trials indicated that the 2017 BSR DI scores were significantly correlated (ρ = 0.19, p < 0.05) with the 2019 greenhouse BSR evaluation data (**Table 2**).

**Table 3:** Analysis of variance for Sclerotinia BSR disease rating (DR) and area under the disease progress curve (AUDPC) values among lines of the HA 89/*H. petiolaris* AB-QTL population determined in greenhouse trials.

Trait	Component	df	Variance estimate	Confidence limit (0.05)		*F*/*Z* value†	*p*-value > *F*/*Z*
				Lower	Upper		
AUDPC	Genotype	173	$\sigma_{g}^{2}\;$ = 2949.27	2,130.38	4,353.59	**2.60**	**<0.0001**
	Rep	2	$\sigma_{e}^{2\;}$ = 40589	10,996	1,611,099	1.00	0.1588
	Error	346	$\sigma_{e}^{2\;}$ = 5515.33	4,777.62	6,439.12		
DR	Genotype	173	$\sigma_{g}^{2}\;$ = 0.2941	0.2118	0.436	**2.57**	**<0.0001**
	Rep	2	$\sigma_{r}^{2}\;$ = 4.0743	1.1037	161.73	1.00	0.1588
	Error	346	$\sigma_{e}^{2\;}$ = 0.5628	0.4875	0.657		

† Data were analyzed using the PROC MIXED model with genotypes considered as a fixed effect and, thus, subject to F-test (values indicated in bold). F, Fisher’s F-test statistic; Z, Z-test statistic; Gen, genotype; Env, environment.

### 3.3 Construction of linkage map

A total of 1,150 SNP markers were mapped to 1,121 unique loci across the 17 sunflower chromosomes (**Table 4**). Out of the total 1,150 SNP markers, only 2.5% (29 SNPs) were co-segregating markers mapped to the same loci as other SNPs in the LGs. The overall length of all 17 LGs covered 997.51 cM with a mean density of 0.89 cM^−1^ Locus and 0.87 cM^−1^ Marker in the HA 89/*H. petiolaris* sunflower linkage map. Considerable variation was observed in the number of mapped loci across the 17 LGs. LG4 exhibited the largest number of mapped loci (161), followed by LG8 (141) and LG9 (121), each having over 100 mapped loci. Five LGs had fewer than 30 loci mapped in the current HA 89/*H. petiolaris* linkage map with the lowest on LG7 (21), followed by LG6 (23), LG3 (24), and LGs 5 and 17 (26 each). The density of the mapped loci ranged from 0.35 to 3.47 cM^−1^ Locus on LG4 and LG6, respectively, across the sunflower genome. The length of individual LGs in the HA 89/*H. petiolaris* linkage map did not show much variation with the longest being LG13 (67.830 cM) while the shortest was LG3 (48.054 cM). A total of fifteen 5- to 10-cM-long gaps between two adjacent loci were observed throughout the genome in the linkage map with a maximum of four on LG7. There were only four gaps *>*10 cM between two adjacent loci with two on LG6 and one each on LGs 12 and 13 (**Table 4**). A more detailed description of the HA 89/*H. petiolaris* linkage map with marker names and map positions is presented in **Supplementary Table S3**.

**Table 4:** Overview of the sunflower linkage map developed using 1,150 SNP markers for an advanced backcross population derived from the cross of inbred line HA 89 with the wild annual species *H. petiolaris.*

Linkage group	Map length (cM)	No. of loci	No. of markers	cM/Locus	cM/Marker	No. of large gaps	
						5–10 cM	>10 cM
LG1	63.395	46	57	1.38	1.11	0	0
LG2	56.017	85	85	0.66	0.66	0	0
LG3	48.054	24	29	2.00	1.66	1	0
LG4	56.641	161	161	0.35	0.35	1	0
LG5	57.998	26	29	2.23	2.00	1	0
LG6	79.824	23	30	3.47	2.66	1	2
LG7	58.503	21	24	2.79	2.44	4	0
LG8	56.214	141	141	0.40	0.40	1	0
LG9	57.210	121	121	0.47	0.47	0	0
LG10	49.143	56	56	0.88	0.88	0	0
LG11	49.535	40	40	1.24	1.24	0	0
LG12	62.845	66	66	0.95	0.95	1	1
LG13	67.830	98	98	0.69	0.69	0	1
LG14	55.900	36	36	1.55	1.55	2	0
LG15	58.807	91	91	0.65	0.65	0	0
LG16	55.684	60	60	0.93	0.93	1	0
LG17	63.909	26	26	2.46	2.46	2	0
Total	997.509	1,121	1150	0.89	0.87	15	4

### 3.4 Analysis of quantitative trait loci associated with BSR resistance

**Figure 2:**
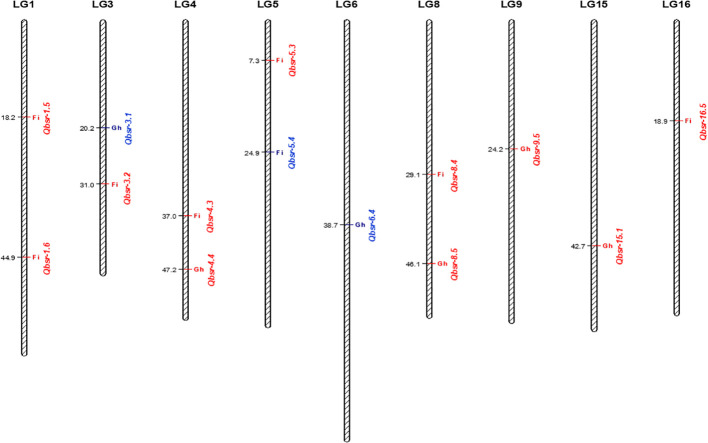
Quantitative trait loci (QTL) associated with Sclerotinia BSR resistance mapped using the HA 89/*H. petiolaris* advanced backcross population. QTL displayed in red font have favorable alleles from *H. petiolaris* and QTL displayed in blue font have favorable alleles from HA 89. Fi, field; Gh, greenhouse.

QTL analysis for the HA 89/*H. petiolaris* AB-QTL population identified a total of 14 genomic regions on nine chromosomes associated with BSR resistance (**Table 5**, **Figure 2**). Each of the chromosomes 1, 3, 4, 5, and 8 had two genomic regions where BSR resistance QTL were detected, while each of the remaining four chromosomes 6, 9, 15, and 16 had one QTL mapped in this population. Eight QTL, *Qbsr-1.5, Qbsr-1.6, Qbsr-3.2, Qbsr-4.3, Qbsr-5.3, Qbsr-5.4, Qbsr-8.4*, and *Qbsr-16.5*, were detected in the field trials measured for BSR DI, while the remaining six QTL, *Qbsr-3.1, Qbsr-4.4, Qbsr-6.4, Qbsr-8.5, Qbsr-9.5*, and *Qbsr-15.1*, were detected in the greenhouse trials measured for AUDPC and/or DR traits. No common genomic region was observed where QTL were detected for both field and greenhouse trials. Eleven QTL had favorable BSR resistance alleles derived from the wild *H. petiolaris* parent, while only three QTL, *Qbsr-3.1, Qbsr-5.4*, and *Qbsr-6.4*, had favorable alleles contributed by HA 89, the susceptible cultivated sunflower parent. *Qbsr-3.2, Qbsr-5.3*, and *Qbsr5-4* were only detected by individual analysis of the 2018 field trial data while *Qbsr-16.5* was only detected by individual analysis of the 2017 field trial data (**Supplementary Table S4**). The phenotypic variance (*R* ^2^) explained by each of the identified QTL varied widely and ranged from 3.5% for *Qbsr-9.5* to as high as 28.1% for *Qbsr-1.5* in this AB-QTL population. A more detailed description of these QTL is presented in **Supplementary Table S4**.

**Table 5:** Sclerotinia BSR resistance QTL identified in the HA 89/*H. petiolaris* AB-QTL population.

QTL	Environment	Linkage group	Position (cM)	LOD range	Flanking markers		*R* ^2^ range	Resistance allele source
					Left	Right		
***Qbsr-1.5***	Field	1	18.2	8.19–14.14	C1_141837870	C1_142468436	19.4–28.1	*H. petiolaris*
***Qbsr-1.6***	Field	1	44.9	7.05	C1_149054858	C1_149054548	13.0	*H. petiolaris*
***Qbsr-3.1***	GH	3	20.2	5.45–5.60	C3_9558059	C3_8189204	7.6–7.9	HA 89
***Qbsr-3.2***	Field	3	31.0	4.08	C3_9948709	C3_10932604	7.7	*H. petiolaris*
***Qbsr-4.3***	Field	4	37.0	6.57–15.70	C4_68555521	C4_53509559	8.3–27.3	*H. petiolaris*
***Qbsr-4.4***	GH	4	47.2	8.93–9.09	C4_41522476	C4_31291162	14.1–14.2	*H. petiolaris*
***Qbsr-5.3***	Field	5	7.3	6.07	C5_14302963	C5_13859353	13.8	*H. petiolaris*
***Qbsr-5.4***	Field	5	24.9	6.04	C5_210306872	C5_211763161	12.0	HA 89
***Qbsr-6.4***	GH	6	38.7	4.68	C6_24249224	C6_20453198	6.3–6.4	HA 89
***Qbsr-8.4***	Field	8	29.1	4.06–5.36	C8_53905152	C8_69977196	5.3–9.8	*H. petiolaris*
***Qbsr-8.5***	GH	8	46.1	6.06–6.56	C8_117061683	C8_114686800	8.1–8.9	*H. petiolaris*
***Qbsr-9.5***	GH	9	24.2	5.19	C9_189181326	C9_189017831	3.5	*H. petiolaris*
***Qbsr-15.1***	GH	15	42.7	6.63–7.15	C15_167594058	C15_114250240	10.1–11.1	*H. petiolaris*
***Qbsr-16.5***	Field	16	18.9	5.79	C16_81804237	C16_54855620	15.2	*H. petiolaris*

LOD, log of odds; R^2^, phenotypic variation explained; GH, greenhouse.

## 4 Discussion

The crop wild relatives (CWR) of sunflower represents a precious source of valuable genes for many biotic and abiotic stress traits (for review, see Seiler et al., 2017b). Although much of this contribution has been derived from the use of major genes, for example, CMS PET1, in herbicide resistance, rust, and downy disease resistances, its use in Sclerotinia disease resistance has been rather limited. The current research is an attempt to characterize BSR resistance QTL introduced from the wild sunflower species *H. petiolaris*. Gene introgression from wild ancestors into domesticated species is often associated with several inherent problems, for example, cross incompatibility, reduced fertility, recombination suppression, and linkage drag (Grandillo and Tanksley, 2005). Tanksley and Nelson (1996) proposed the AB-QTL strategy to simultaneously identify favorable QTL alleles in the genome of wild species and to transfer them into a cultivated background for elite germplasm development. In the present study, we successfully used an AB-QTL population to identify BSR resistance QTL derived from the wild sunflower species *H. petiolaris*. Of 14 QTL detected, 11 had favorable resistance alleles from the *H. petiolaris* parent.

The prevalence of BSR disease in all three field seasons was comparable with mean BSR DI ranging between 47.4% and 51.9%. The distribution of BSR DI data showed continuous patterns around the mean values in all three seasons consistent with a quantitative trait where resistance is conferred by many small effect genes with additive effect (Young, 1996). Interestingly, the distribution of mean BSR DI data in the three seasons was skewed toward lower values, indicating that some progeny lines showed differential responses in different growing seasons. However, the nonsignificant genotype × environment interaction (*p* = 0.100) for the combined ANOVA suggests that genotypes responded similarly across trial years and there were no significant crossover interactions having a large effect on rank order of genotypes between trial years. In the greenhouse trial, BSR resistance was evaluated using a different set of evaluation parameters (AUDPC and DR) for the population. Despite different BSR evaluation parameters used in the greenhouse trial, both AUDPC and DR also showed continuous variations in the population, further confirming that BSR resistance is a quantitative trait. Contrary to the BSR DI response observed in the field, both AUDPC and DR observations were skewed towards higher susceptible values in the greenhouse trial. These results are not surprising since the AB-QTL population investigated was derived from a BC_2_ progeny line, and theoretically, this population is expected to have recovered 87.5% of the cultivated sunflower parent genome, which is susceptible to BSR. Similar trends were also observed when sunflower AB-QTL populations derived from *H. argophyllus* and *H. praecox* were tested in both field and greenhouse environments (Talukder et al., 2021; Talukder et al., 2022).

The apparent inconsistency observed in the BSR disease response in the field and greenhouse trials might have resulted from the inoculation technique used in these two different disease screening environments. In the field trials, the *S. sclerotiorum*-infested millet inoculum was side dressed in furrows placed 15–20 cm from the base of the plants, while the plants were inoculated in the greenhouse by placing inoculum in direct contact with roots. Owing to the distant placement of the inoculum source from the root system in the field trials, some of the plants may have avoided being infected by the pathogen, which is not possible in the greenhouse trial. In addition, unpredictable weather conditions such as soil moisture and temperature during field testing may have affected disease development. The correlations between BSR evaluation traits measured in the greenhouse and field evaluations across seasons were also inconsistent and only showed a significant (*p* < 0.05) positive relationship for the 2017 field season. Nevertheless, despite the disparity in BSR response observed between field and greenhouse trials, the broad-sense heritability (*H* ^2^) estimates were the same (61%) for both the environments, suggesting a similar contribution of the genetic makeup to the observed phenotype in both environments. It is likely that the field and greenhouse trials measure somewhat distinct aspects of resistance to this disease and, thus, may provide complementary information.

Linkage analysis placed a total of 1,150 SNP markers to 1,121 genetic loci across 17 sunflower chromosomes. The total linkage map spans only 997.51 cM, which is significantly shorter than the two previously published interspecific linkage maps developed from wild annual species, *H. argophyllus* and *H. praecox* (Talukder et al., 2021; Talukder et al., 2022). There are previous reports indicating that interspecific hybridization can result in suppressed recombination and reduced map size compared with observations from intraspecific hybridization (Gebhardt et al., 1991; Causse et al., 1994; Ky et al., 2000; Torello Marinoni et al., 2020). Fewer recombination events during population development, coupled with the exclusion of a large number of highly distorted markers during mapping analysis, might have caused such a short linkage map for this HA 89/*H. petiolaris* AB-QTL population. Nonetheless, the locus density per cM of the map was higher with a lower frequency of large gaps in the linkage map than observed for previous interspecific linkage maps.

QTL analysis revealed a total of 14 genomic regions associated with BSR resistance located on nine sunflower chromosomes. The QTL detected on LG3 and LG15 are unique since no BSR resistance QTL introgressions have previously been reported on these two chromosomes. Eight QTL were identified for BSR DI determined in field trials and six QTL were detected using data from greenhouse tests. Surprisingly, none of these QTL were common to both the field and greenhouse environments, verifying the low correlations observed between the phonotypic traits in the two environments. As expected, approximately 79% of the QTL identified using this AB-QTL population have favorable alleles conferring BSR resistance derived from the wild *H. petiolaris* parent. This result is consistent with our previous reports where 62% and 84% of the alleles conferring BSR resistance in AB-QTL populations were derived from the wild *H. argophyllus* and *H. praecox* parents, respectively (Talukder et al., 2021; Talukder et al., 2022). In general, the QTL with resistance conferring alleles derived from the *H. petiolaris* parent explained comparatively greater proportions of the phenotypic variation (mean *R* ^2^, 13%) in the AB-QTL population than those QTL where resistance alleles were derived from the cultivated sunflower parent (mean *R* ^2^, 8%) (**Supplementary Table S4**). Four of the detected BSR resistance QTL were identified by individual analysis of single year field trial data and were not detected in combined analysis or across multiple years of the field trials, suggesting that the resistance conferred by these QTL is affected by environmental conditions and these QTL may not provide stable resistance under differing conditions or years.

Several studies have been reported for BSR QTL analysis in sunflower (Davar et al., 2010; Amouzadeh et al., 2013; Talukder et al., 2014b; Talukder et al., 2016; Paknia et al., 2020; Talukder et al., 2021; Talukder et al., 2022). A comparative analysis would be interesting to find out whether the QTL identified in this study are the same as those that have been previously reported. However, some of the reported QTL in previous studies lacked detailed physical location on the sunflower genome or associated marker sequence information for comparison. Comparing the physical positions of the QTL mapped in the current research with those mapped in prior studies revealed that most of the QTL identified in this study are novel and have not been previously detected in other populations with the exceptions of *Qbsr-8.4* on LG8 and *Qbsr-16.5* on LG16 (**Supplementary Table S5**). The genomic location of the *Qbsr-8.4* QTL overlapped with the genomic locations of the *Qbsr-8.1* and *Qbsr-8.2* QTL mapped in the HA 89/*H. argophyllus* AB-QTL population, and also the *Qbsr-8.3* QTL mapped in the HA 89/*H. praecox* AB-QTL population (Talukder et al., 2021; Talukder et al., 2022). Similarly, *Qbsr-16.5* mapped on LG16 in this study was located in the same genomic region as *Qbsr-16.4* in the HA 89/*H. argophyllus* AB-QTL population (Talukder et al., 2021). Further studies will be required to determine if the same causal genes are responsible for BSR resistance among the different populations at these co-located QTL. However, it is interesting to note that the wild sunflower accessions used as parents in these studies were all collected from the southwestern United States, with the *H. argophyllus* and *H. praecox* accessions collected from Texas and the *H. petiolaris* accession used in this study collected in New Mexico.

Traditionally, Sclerotinia QTL mapping studies in sunflower have been centered within the elite gene pool of the crop, thus reducing the probability of identifying novel alleles that can be used for genetic improvement of elite germplasm due to the narrow genetic base of cultivated *H. annuus*. Searching for novel sources of resistance and mapping QTL with high-throughput DNA markers have been the central focus of our Sclerotinia resistance research. Different mapping populations were developed using both intraspecific and interspecific crosses and have resulted in the mapping of BSR resistance QTL on all 17 sunflower chromosomes along with dissemination of the necessary details to deploy them into elite sunflower lines using marker-assisted selection breeding. These prior efforts, along with those described in the current study, provide valuable genetic diversity for BSR resistance that can be employed by sunflower breeders to improve resistance of hybrids to this important disease.

## Data availability statement

The datasets presented in this study can be found in online repositories. The names of the repository/repositories and accession number(s) can be found below: https://www.ebi.ac.uk/eva/?evastudy=PRJEB65096, PRJEB65096.

## Author contributions

ZT: Conceptualization, Formal Analysis, Investigation, Writing – original draft. WU: Formal Analysis, Funding acquisition, Investigation, Writing – review & editing. CM: Investigation, Writing – review & editing. XL: Investigation, Project administration, Writing – review & editing. GS: Investigation, Resources, Writing – review & editing. XC: Investigation, Project administration, Writing – review & editing. LQ: Conceptualization, Formal Analysis, Funding acquisition, Investigation, Project administration, Resources, Supervision, Writing – review & editing.

## Supplementary material

Supplementary Figure 1 Heat map summarizing BSR response of 174 individuals of the HA 89/*H. petiolaris* advanced backcross population determined in field and greenhouse trials. Area under the disease progress curve (AUDPC) and disease rating (DR) were determined in greenhouse trials in 2019 while disease incidences (DI) were determined in field trails at Carrington, ND during 2017 - 2019.

## References

[B1] Amouzadeh M. Darvishzadeh R. Haddadi P. Abdollahi M. B. Rezaee D. Y. (2013). Genetic analysis of partial resistance to basal stem rot (*Sclerotinia sclerotiorum*) in sunflower. Genetika 45, 737–748. doi: 10.2298/GENSR1303737A

[B2] Badouin H. Gouzy J. Grassa C. J. Murat F. Staton S. E. Cottret L.. (2017). The sunflower genome provides insights into oil metabolism, flowering and Asterid evolution. Nature 546, 148–152. doi: 10.1038/nature22380 28538728

[B3] Bert P. F. Jouan I. De Labrouhe D. T. Serre F. Nicolas P. Vear F. (2002). Comparative genetic analysis of quantitative traits in sunflower (*Helianthus annuus* L.) 1. QTL involved in resistance to *Sclerotinia sclerotiorum* and *Diaporthe helianthi* . Theor. Appl. Genet. 105, 985–993. doi: 10.1007/s00122-002-1004-3 12582925

[B4] Blackman B. K. Scascitelli M. Kane N. C. Luton H. H. Rasmussen D. A. Bye R. A.. (2011). Sunflower domestication alleles support single domestication center in eastern North America. Proc. Natl. Acad. Sci. U.S.A. 108, 14360–14365. doi: 10.1073/pnas.1104853108 21844335 PMC3161615

[B5] Block C. C. Marek L. F. Gulya T. J. (2010). Evaluation of wild *Helianthus* species for resistance to Sclerotinia stalk rot, in Proceedings of the 8th annual sclerotinia initiative meeting (Bloomington, MN, USA (Fargo, ND: USDA-ARS National Sclerotinia Initiative), 12.

[B6] Block C. C. Marek L. F. Gulya T. J. (2011). “Evaluation of wild *Helianthus* species for resistance to Sclerotinia stalk rot,” in Proceedings of the 9th annual sclerotinia initiative meeting (Bloomington, MN, USA (Fargo, ND: USDA-ARS National Sclerotinia Initiative), 13.

[B7] Boland G. Hall R. (1994). Index of plant hosts of *Sclerotinia sclerotiorum* . Can. J. Plant Pathol. 16, 93–108. doi: 10.1080/07060669409500766

[B8] Bolton M. D. Thomma B. P. Nelson B. D. (2006). *Sclerotinia sclerotiorum* (Lib.) de Bary: biology and molecular traits of a cosmopolitan pathogen. Mol. Plant Pathol. 7, 1–16. doi: 10.1111/j.1364-3703.2005.00316.x 20507424

[B9] Cáceres C. Castaño F. Rodríguez R. Ridao A. Salaberry T. Echeverría M.. (2006). Variability of responses of *Helianthus petiolaris* to *Sclerotinia sclerotiorum* infections. Helia 29, 43–48. doi: 10.2298/HEL0645043C

[B10] Causse M. A. Fulton T. M. Cho Y. G. Ahn S. N. Chunwongse J. Wu K.. (1994). Saturated molecular map of the rice genome based on an interspecific backcross population. Genetics 138, 1251–1274. doi: 10.1093/genetics/138.4.1251 7896104 PMC1206261

[B11] Cheres M. T. Knapp S. J. (1998). Ancestral origins and genetic diversity of cultivated sunflower: coancestry analysis of public germplasm. Crop Sci. 38, 1476–1482. doi: 10.2135/cropsci1998.0011183X003800060012x

[B12] Churchill G. A. Doerge R. W. (1994). Empirical threshold values for quantitative trait mapping. Genetics 138, 963–971. doi: 10.1093/genetics/138.3.963 7851788 PMC1206241

[B13] Core Team. R. (2021). R: A language and environment for statistical computing (Vienna, Austria: R Foundation for Statistical Computing). Available at: http://www.R-project.org/.

[B14] Davar R. Darvishzadeh R. Majd A. Ghosta Y. Sarrafi A. (2010). QTL mapping of partial resistance to basal stem rot in sunflower using recombinant inbred lines. Phytopathol. Mediterr. 49, 330–341. doi: 10.14601/Phytopathol_Mediterr-8374

[B15] Elshire R. J. Glaubitz J. C. Sun Q. Poland J. A. Kawamoto K. Buckler E. S.. (2011). A robust, simple genotyping-by-sequencing (GBS) approach for high diversity species. PloS One 6, e19379. doi: 10.1371/journal.pone.0019379 21573248 PMC3087801

[B16] Gebhardt C. Ritter E. Barone A. Debener T. Walkemeier B. Schachtschabel U.. (1991). RFLP maps of potato and their alignment with the homoeologous tomato genome. Theor. Appl. Genet. 83, 49–57. doi: 10.1007/BF00229225 24202256

[B17] Grandillo S. Tanksley S. D. (2005). “Advanced backcross QTL analysis: Results and perspectives,” in Proceedings of the International Congress, 'In the wake of the double helix: from the green revolution to the gene revolution'. Eds. Tuberosa R. Phillips R. L. Gale. M. (Bologna, Italy: Avenue Media), 115–132.

[B18] Gulya T. J. Buetow R. Kandel H. (2018). “2017 NSA sunflower survey: disease incidence,” in Proceedings of the 40th sunflower research forum (Fargo, ND, USA: Manden, ND: National Sunflower Association).

[B19] Gulya T. J. Harveson R. Mathew F. Block C. C. Thompson S. Kandel H.. (2019). Comprehensive disease survey of U.S. sunflower: disease trends, research priorities and unanticipated impacts. Plant Dis. 103, 601–618. doi: 10.1094/PDIS-06-18-0980-FE 30789318

[B20] Gulya T. J. Radi S. Balbyshev N. (2008). “Large scale field evaluations for Sclerotinia stalk rot resistance in cultivated sunflower,” in Proceedings of the 17th international sunflower conference. Ed. Velasco L. (Córdoba, Spain: Paris, France: International Sunflower Association), 175–179.

[B21] Gulya T. J. Rashid K. Y. Masirevic S. M. (1997). “Sunflower diseases,” in Sunflower technology and production. Ed. Schneiter. A. A. (Madison, WI, USA: ASA-CSSA-SSSA), 263–379.

[B22] Gulya T. J. Vick B. A. Nelson B. D. (1989). Sclerotinia head rot of sunflower in North Dakota: 1986 incidence, effect on yield and oil components, and sources of resistance. Plant Dis. 73, 504–507. doi: 10.1094/PD-73-0504

[B23] Gutierrez A. Cantamutto M. Poverene M. (2012). Disease tolerance in *Helianthus petiolaris*: a genetic resource for sunflower breeding. Plant Product Sci. 15, 204–208. doi: 10.1626/pps.15.204

[B24] Horne E. C. Kumpatla S. P. Patterson K. A. Gupta M. Thompson S. A. (2004). Improved high-throughput sunflower and cotton genomic DNA extraction and PCR fidelity. Plant Mol. Biol. Rep. 22, 83–84. doi: 10.1007/BF02773352

[B25] Hulke B. S. Ma G. Qi L. L. Gulya T. J. (2018). Registration of oilseed sunflower germplasms RHA 461, RHA 462, RHA 463, HA 465, HA 466, HA 467, and RHA 468 with diversity in Sclerotinia resistance, yield, and other traits. J. Plant Regist. 12, 142–147. doi: 10.3198/jpr2017.04.0023crg

[B26] Jan C.-C. Quresh Z. Gulya T. (2004). Registration of seven rust resistant sunflower germplasms. Crop Sci. 44, 1887–1889. doi: 10.2135/cropsci2004.1887a

[B27] Jan C.-C. Rutger J. N. (1988). Mitomycin C- and Streptomycin-induced male sterility in cultivated sunflower. Crop Sci. 28, 792–795. doi: 10.2135/cropsci1988.0011183X002800050014x

[B28] Joehanes R. Nelson J. C. (2008). QGene 4.0, an extensible Java QTL-analysis platform. Bioinformatics 24, 2788–2789. doi: 10.1093/bioinformatics/btn523 18940826

[B29] Koehler B. D. Gulya T. J. Hulke B. S. (2019). Registration of oilseed sunflower germplasms RHA 478, RHA 479, RHA 480, and HA 481 providing diversity in resistance to necrotrophic pathogens of sunflower. J. Plant Regist. 13, 444–449. doi: 10.3198/jpr2019.04.0017crg

[B30] Kosambi D. D. (1943). The estimation of map distances from recombination values. Ann. Eugen. 12, 172–175. doi: 10.1111/j.1469-1809.1943.tb02321.x

[B31] Ky C.-L. Barre P. Lorieux M. Trouslot P. Akaffou S. Louarn J.. (2000). Interspecific genetic linkage map, segregation distortion and genetic conversion in coffee (Coffea sp.). Theor. Appl. Genet. 101, 669–676. doi: 10.1007/s001220051529

[B32] Ladizinsky G. (1985). Founder effect in crop-plant evolution. Econ. Bot. 39, 191–199. doi: 10.1007/BF02907844

[B33] Leclercq P. (1969). Une sterilite male cytoplasmique chez le tournesol. Ann. Amel. Plantes 19, 99–106.

[B34] Meng L. Li H. Zhang L. Wang J. (2015). QTL IciMapping: integrated software for genetic linkage map construction and quantitative trait locus mapping in biparental populations. Crop J. 3, 269–283. doi: 10.1016/j.cj.2015.01.001

[B35] Miller J. F. Gulya T. J. (1999). Registration of eight Sclerotinia-tolerant sunflower germplasm lines. Crop Sci. 39, 301–302. doi: 10.2135/cropsci1999.0011183X003900010075x

[B36] Money K. L. Koehler B. D. Misar C. G. Grove M. Underwood W. Hulke B. S. (2019). Registration of oilseed sunflower germplasms RHA 485, RHA 486, and HA 487, selected for resistance to Phomopsis stalk canker and Sclerotinia, in a high-yielding and high-oil background. J. Plant Regist. 13, 439–442. doi: 10.3198/jpr2019.02.0008crg

[B37] Nyquist W. E. (1991). Estimation of heritability and prediction of selection response in plant populations. Crit. Rev. Plant Sci. 10, 235–322. doi: 10.1080/07352689109382313

[B38] Paknia R. Darvishzadeh R. Shahriari F. Malekzadeh S. Maleki H. H. (2020). Analyses of genomic regions linked with resistance to basal stem rot in sunflower (*Helianthus annuus* L.) under field conditions. Iran. J. Genet. Plant Breed. 9, 41–50. doi: 10.30479/ijgpb.2020.13761.1277

[B39] Qi L. L. Hulke B. S. Vick B. A. Gulya T. J. (2011). Molecular mapping of the rust resistance gene *R_4_ * to a large NBS-LRR cluster on linkage group 13 of sunflower. Theor. Appl. Genet. 123, 351–358. doi: 10.1007/s00122-011-1588-6 21479933

[B40] Qi L. L. Long Y. M. Jan C. C. Ma G. J. Gulya T. J. (2015). *Pl* _17 is a_ novel gene independent of known downy mildew resistance genes in the cultivated sunflower (*Helianthus annuus* L.). Theor. Appl. Genet. 128, 757–767. doi: 10.1007/s00122-015-2470-8 25673143

[B41] Qi L. L. Long Y. M. Talukder Z. I. Seiler G. J. Block C. C. Gulya T. J. (2016). Genotyping-by-sequencing uncovers the introgression alien segments associated with Sclerotinia basal stalk rot resistance from wild species-I. *Helianthus argophyllus* and *H. petiolaris* . Front. Genet. 7. doi: 10.3389/fgene.2016.00219 PMC5183654 28083014

[B42] Qi L. L. Talukder Z. I. Long Y. M. Seiler G. J. (2018). Registration of oilseed sunflower germplasms HA-BSR2, HA-BSR3, HA-BSR4, and HA-BSR5 with resistance to Sclerotinia basal stalk rot and downy mildew. J. Plant Regist. 12, 399–404. doi: 10.3198/jpr2017.11.0083crg

[B43] Rieseberg L. H. Kim M. J. Seiler G. J. (1999). Introgression between the cultivated sunflower and a sympatric wild relative, *Helianthus petiolaris* (Asteraceae). Int. J. Plant Sci. 160, 102–108. doi: 10.1086/314107

[B44] Rogers C. E. Thompson T. E. Seiler G. J. (1984). Registration of three *Helianthus* germplasm lines for resistance to the sunflower moth. Crop Sci. 24, 212–213. doi: 10.2135/cropsci1984.0011183X002400010058x

[B45] SAS Institute. (2016). The SAS system for windows (Cary, NC, USA: SAS Institute Inc.).

[B46] Schneiter A. A. Miller J. F. (1981). Description of sunflower growth stages. Crop Sci. 21, 901–903. doi: 10.2135/cropsci1981.0011183X002100060024x

[B47] Seiler G. J. (2010). “Utilization of wild *Helianthus* species in breeding for disease resistance,” in Proceedings of the International Symposium “Sunflower breeding on resistance to diseases (Krasnodar, Russia: Paris, France: International Sunflower Association), 37–51.

[B48] Seiler G. J. Misar C. G. Gulya T. J. Underwood W. R. Flett B. C. Gilley M. A.. (2017a). Identification of novel sources of resistance to Sclerotinia basal stalk rot in South African sunflower germplasm. Plant Health Prog. 18, 87–90. doi: 10.1094/php-01-17-0007-rs

[B49] Seiler G. J. Qi L. L. Marek L. F. (2017b). Utilization of sunflower crop wild relatives for cultivated sunflower improvement. Crop Sci. 57, 1083–1101. doi: 10.2135/cropsci2016.10.0856

[B50] Smart B. C. Koehler B. D. Misar C. G. Gulya T. J. Hulke B. S. (2019). Registration of oilseed sunflower germplasms HA 482, RHA 483, and RHA 484 selected for resistance to Sclerotinia and Phomopsis diseases. J. Plant Regist. 13, 450–454. doi: 10.3198/jpr2019.07.0030crg

[B51] Stam P. (1993). Construction of integrated genetic linkage maps by means of a new computer package: Join Map. Plant J. 3, 739–744. doi: 10.1111/j.1365-313X.1993.00739.x

[B52] Talukder Z. I. Hu J. Seiler G. J. Qi L. L. (2017). Registration of oilseed sunflower germplasm HA-BSR1 highly tolerant to Sclerotinia basal stalk rot. J. Plant Regist. 11, 315–319. doi: 10.3198/jpr2016.10.0060crg

[B53] Talukder Z. I. Hulke B. S. Marek L. F. Gulya T. J. (2014a). Sources of resistance to sunflower diseases in a global collection of domesticated USDA plant introductions. Crop Sci. 54, 694–705. doi: 10.2135/cropsci2013.07.0506

[B54] Talukder Z. I. Hulke B. S. Qi L. L. Scheffler B. E. Pegadaraju V. Mcphee K.. (2014b). Candidate gene association mapping of Sclerotinia stalk rot resistance in sunflower (*Helianthus annuus* L.) uncovers the importance of *COI*1 homologs. Theor. Appl. Genet. 127, 193–209. doi: 10.1007/s00122-013-2210-x 24193356

[B55] Talukder Z. I. Long Y. M. Seiler G. J. Underwood W. Qi L. L. (2019a). Introgression and monitoring of wild *Helianthus praecox* alien segments associated with Sclerotinia basal stalk rot resistance in sunflower using genotyping-by-sequencing. PloS One 14, e0213065. doi: 10.1371/journal.pone.0213065 30822322 PMC6396933

[B56] Talukder Z. I. Long Y. M. Seiler G. J. Underwood W. Qi L. L. (2019b). Registration of oilseed sunflower germplasms HA-BSR6, HA-BSR7, and HA-BSR8 highly resistant to Sclerotinia basal stalk rot and downy mildew. J. Plant Regist. 13, 433–438. doi: 10.3198/jpr2018.10.0071crg

[B57] Talukder Z. I. Seiler G. J. Song Q. Ma G. Qi L. L. (2016). and QTL mapping of Sclerotinia basal stalk rot resistance in sunflower using genotyping-by-sequencing. Plant Genome 9, 35. doi: 10.3835/plantgenome2016.03.0035 27902793

[B58] Talukder Z. I. Underwood W. Misar C. G. Seiler G. J. Cai X. Li X.. (2022). Genomic insights into Sclerotinia basal stalk rot resistance introgressed from wild *Helianthus praecox* into cultivated sunflower (*Helianthus annuus* L.). Front. Plant Sci. 13. doi: 10.3389/fpls.2022.840954 PMC9158519 35665155

[B59] Talukder Z. I. Underwood W. Misar C. G. Seiler G. J. Liu Y. Li X.. (2021). Unraveling the Sclerotinia basal stalk rot resistance derived from wild *Helianthus argophyllus* using a high-density single nucleotide polymorphism linkage map. Front. Plant Sci. 11. doi: 10.3389/fpls.2020.617920 PMC7886805 33613588

[B60] Tanksley S. Nelson J. (1996). Advanced backcross QTL analysis: a method for the simultaneous discovery and transfer of valuable QTLs from unadapted germplasm into elite breeding lines. Theor. Appl. Genet. 92, 191–203. doi: 10.1007/BF00223376 24166168

[B61] Torello Marinoni D. Nishio S. Valentini N. Shirasawa K. Acquadro A. Portis E.. (2020). Development of high-density genetic linkage maps and identification of loci for chestnut gall wasp resistance in castanea spp. Plants 9, 1048. doi: 10.3390/plants9081048 32824716 PMC7465717

[B62] Underwood W. Misar C. G. Block C. C. Gulya T. J. Talukder Z. I. Hulke B. S.. (2021). A greenhouse method to evaluate sunflower quantitative resistance to basal stalk rot caused by *Sclerotinia sclerotiorum* . Plant Dis. 105, 464–472. doi: 10.1094/pdis-08-19-1790-re 33264029

[B63] USDA. (2022). United states department of agriculture, national agricultural statistics service (Washington, DC USDA). Available at: https://quickstats.nass.usda.gov/results/43B1B618-6E4C-3B15-A426-7E8202E3B950 (Accessed June 3, 2023).

[B64] USDA. (2023). Oilseeds: world markets and trade (United States: Department of Agriculture). Available at: https://apps.fas.usda.gov/psdonline/circulars/oilseeds.pdf (Accessed June 3, 2023).

[B65] Van Ooijen J. (2006). JoinMap® 4, Software for the calculation of genetic linkage maps in experimental populations. Kyazma BV Wageningen, Netherlands.

[B66] Voorrips R. E. (2002). MapChart: software for the graphical presentation of linkage maps and QTLs. J. Hered. 93, 77–78. doi: 10.1093/jhered/93.1.77 12011185

[B67] Wang S. Basten C. J. Zeng Z. B. (2012). Windows QTL cartographer (Raleigh, NC: Department of Statistics, North Carolina State University). Available at: http://statgen.ncsu.edu/qtlcart/WQTLCart.htm (Accessed June 25, 2023).

[B68] Young N. (1996). QTL mapping and quantitative disease resistance in plants. Annu. Rev. Phytopathol. 34, 479–501. doi: 10.1146/annurev.phyto.34.1.479 15012553

[B69] Zeng Z. B. (1994). Precision mapping of quantitative trait loci. Genetics 136, 1457–1468. doi: 10.1093/genetics/136.4.1457 8013918 PMC1205924

