# Supplementary figures and images for: Genetic analysis of basal stalk rot resistance introgressed from wild Helianthus petiolaris into cultivated sunflower (Helianthus annuus L.) using an advanced backcross population
Source: Front Plant Sci. 2023 Oct 18;14:1278048. doi: 10.3389/fpls.2023.1278048 (PMC10619160; doi:10.3389/fpls.2023.1278048)

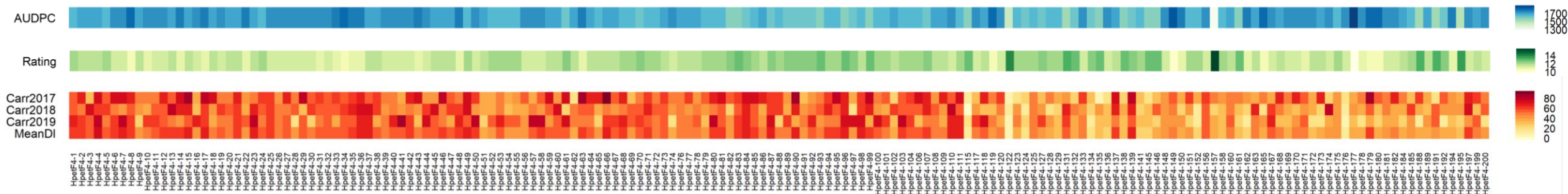

Advanced backcross progeny lines

Supplement: Supplementary Figure 1 — Heat map summarizing BSR response of 174 individuals of the HA 89/H. petiolaris advanced backcross population determined in field and greenhouse trials. Area under the disease progress curve (AUDPC) and disease rating (DR) were determined in greenhouse trials in 2019 while disease incidences (DI) were determined in field trails at Carrington, ND during 2017 - 2019. [file DataSheet_1.pdf]
